# Birth, attitudes and placentophagy: a thematic discourse analysis of discussions on UK parenting forums

**DOI:** 10.1186/s12884-020-2824-3

**Published:** 2020-03-06

**Authors:** Riley Botelle, Chris Willott

**Affiliations:** King’s College London, London, UK

**Keywords:** Placentophagy, Thematic analysis, Discourse analysis, Birth, Placenta, Medicalisation, Netnography, Pregnancy

## Abstract

**Background:**

The post-partum consumption of the placenta by the mother (placentophagy) has been practiced since the 1970s in the global North and is seemingly increasing in popularity. Maternal placentophagy is not known to have been practiced in any other time period or culture, despite being near-ubiquitous in other placental mammals. An in-depth qualitative exploration as to the reasons for the practice, its increasing popularity and how it is narratively incorporated into discourses surrounding “ideal” natural and medical births are given in this paper.

**Methods:**

1752 posts from 956 users across 85 threads from the parenting forums Mumsnet and Netmums were identified for inclusion. A thematic discourse analysis was performed using NVivo.

**Results:**

Three main themes were identified: women recounted predominantly positive attitudes towards their own experiences of placentophagy, and they were respectful of others’ views and experiences; some had negative views, particularly around the concept of disgust, but again, they were respectful of others’ experiences. By far the most common method of consumption of the placenta was encapsulation.

**Conclusions:**

This paper identifies the motivation for placentophagy to almost universally be for medical benefits, most commonly the prevention or treatment of post-natal depression (PND). Whilst disgust is a common reaction, discussion of risks is rare, and positive experiences outweigh negative ones. The increasing popularity of the practice is ascribed in part to the comparative palatability of encapsulation and the use of the internet to share resources and remove barriers. Parenting forums are important spaces to negotiate normative birth practices, including placentophagy, and act to build communities of women who value personal experience over medical evidence and highly value personal choice and bodily autonomy. Placentophagy is discussed in terms of its relation to natural and medical births with arguments being made using both discourses for and against the practice. This paper argues that placentophagy is practiced as a resistance to medicalisation as an assertion of control by the mother, whilst simultaneously being a medicalised phenomenon itself.

## Background

Human placentophagy is “the ingestion of a human placenta postpartum, at any time, by any person, in raw or altered form.” [1] Human maternal placentophagy is specifically consumption by the mother of her own placenta. This relatively modern phenomenon is practiced predominantly by white, middle class, married women in the global North [2], and has grown rapidly since the 1970s [3]. When “placentophagy” is used in this paper it can be assumed that it refers to postpartum human maternal placentophagy (the post-birth consumption of the placenta by the person who has given birth) unless otherwise specified.

The method of placentophagy most commonly practiced is “encapsulation”, a process where the placenta is dehydrated, ground and placed into capsules [4]. This is done with either raw or steamed placenta. Alternatively, some consume the placenta raw, often frozen in a smoothie, or cooked, usually in dishes as a substitute for meat.

Whilst prevalence is difficult to estimate, placentophagy is known to be practiced in North America, Oceania and Europe, plus parts of Latin America, the Middle East and Asia and is apparently growing in popularity [5]. In a 2018 study of a medical records dataset for births outside of hospitals containing 23,242 birth events in the United States, 30.8% of mothers consumed their placenta [4]. From 2009 to 2015 Google searches for “placenta encapsulation” increased 100-fold [3]. A search using the internal search engine on YouTube on 25th June 2012 for “placenta encapsulation” yielded 97 results [2]. An identical search on 26th May 2018 yields 5160. In a 2016 internet survey, 66% of participants were aware of placentophagy and 3.3% had practiced it themselves [6]. Media coverage includes TIME, USA Today, New York Magazine, the British Broadcasting Corporation (BBC), and some high-profile celebrities [2].

The self-reported reasons for practicing placentophagy include mood stabilization, enhancing recovery from birth and increasing milk production, although there is no clinical evidence to support any of these. Hypothetical benefits (immunological, hormonal and psychological) have been sporadically suggested in professional literature since the British Medical Journal (BMJ) in 1902, with increasing mention after the 1980s. Hypothetical risks have also been proposed (toxicological, endocrinological and immunological) with little evidence supporting them [1], and a large medical records study showed no association with adverse neonatal outcomes [4]. The exception to this is a case of infant infection potentially associated with placental encapsulation reported in 2016 [7].

The intention of this paper is to research how users of United Kingdom (UK) parenting forums discuss placentophagy online, what this tells us about the practice, and how the interactions between users in these discussions illuminates wider discourses of the interactions between mothering identity, ideal birth scenarios and healthcare. The motivations for placentophagy are especially interesting due to the lack of any historical or cultural record of the practice [8] despite maternal placentophagy being ubiquitous among other placental animals including non-human primates [9]. This is a qualitative paper; for a thorough biomedical review see Farr et al’s 2017 paper [5] and randomised controlled trial (RCT) results by Young et al. [10–12].

### Research aims

This paper explores the discourse produced on two UK internet forums regarding the practice of placentophagy and how opinions are expressed, discussed, debated and created through forum participation. The following questions guide this research:

1. What views do users of UK parenting forums Mumsnet and Netmums express in discourse surrounding participation in placentophagy?
2. What can discussions about placentophagy on these forums tell us about how placentophagy is practiced and why the practice has grown since the 1970s?
3. How do views about placentophagy and interactions between users relate to the macrosocial context (of pregnancy, autonomy, authority and healthcare)?

## Methods

### Methods: thematic and discourse analysis

This research was conducted using thematic discourse analysis applied to posts in forum threads found on two UK-based parenting discussion forums, Mumsnet and Netmums. Thematic analysis is a method for identifying, analysing, and reporting repeated patterns of meaning across a dataset [13] and involves repetitive coding and re-coding of text and aggregation of these codes into larger themes. A “code” is “the most basic segment. .. that can be assessed in a meaningful way regarding the phenomenon” ([14]:p. 63). Themes were reviewed to make sure data within them was coherent, distinct from data in other themes and to make sure they reflected an overview of the data. Themes were then analysed at a deeper level, considering what, if anything, they indicate about broader social discourses in relation to the phenomenon, in part through applying discourse analysis. Discourse analysis is a method that examines how individuals construct their internal understanding of phenomena through discourse [15] and has been suggested to be a particularly useful tool in discussion forum data analysis [16]. The combination of thematic analysis with discourse analysis has been used previously (see for instance Taylor and Ussher [17]; Clarke [18]). As an inductive process, the themes created are strongly linked to the data [19]. This process was assisted by using NVivo, a qualitative data analysis program.

### Discussion forums and analysis

Discussion forums form communities based on common interests and experiences through asynchronous interaction with multiple other users (Montero-Fleta B, Watts F, García-Carbonell A: Discourse analysis of internet-forum communication and its applications to simulation and gaming, unpublished). In these spaces, shared understandings can be created, countered and reaffirmed. Mothers use internet discussion forums to create and disseminate their own personal views about mothering identity, therefore studies of forums offer the opportunity to explore “the dominant ideologies of motherhood” [20]. Each discussion forum contains topic-based sub-forums made up of threads. Threads consist of an initial post made by a user and other users create posts as responses. Discourse production is doubly articulated; it exists both for the active users of the thread and for the “unseen audience” and casual visitors. Users in a thread can read and reflect on others’ contributions before responding, and many users read threads without posting responses at all [21].

Threads were chosen by using the search function on both forums to search for “placentophagy”, “placenta eating”, “placenta encapsulation” and “eat placenta”. Any duplicates or threads not related to the research aims were removed. Threads were converted to plain text and anonymised.

### Choice of forums

The discussion forums chosen for this study (Mumsnet and Netmums)^1^ are open access, meaning all threads are available for the public to read; however only registered users may contribute posts. Mumsnet was established in 2000 and states its aim as to “make parents’ lives easier by pooling knowledge and experience.” There are over 14 million visits to the website each month, over 600,000 registered users and the discussion forum section of the website (“Mumsnet Talk”) has over 25,000 posts each day [22]. Sixty-nine percent of users said they talked about things on Mumsnet that offline friends did not know about [23], highlighting that it may be a useful resource for data regarding potentially taboo practices such as placentophagy. Mumsnet is known to have a demographic skewed towards older, well-educated, middle class women, with a dominance of users from London and South-East England [22, 23]. Given these demographics it is useful to have a second discussion forum to draw data from. The forum chosen for this is Netmums, also established in 2000. Netmums self-reports having a comparatively greater number of users from low income families.^2^ The inclusion of Netmums is in part to assist in creating a more diverse demography of users for discourse production. This is important because parenting is stratified by class [22]. However, it is impossible to know the demographics of users for certain.

### Ethics

The research community has been described as “divided” on the subject of informed consent for the use of material published on open access forums [24]. Seale et al, argue that consent is not needed as posts exist in the public domain [24]. Indeed, Mumsnet have previously published books that have included forum posts in their entirety without seeking direct consent, meaning that users are likely to be aware that their posts may be spread in other formats [28]. However, consciousness of the public ability to view material is not necessarily the same as consenting to be part of research [29]. Due to this the analysis is aggregated into broader themes with no reference to usernames to protect the identities of users. Any quotations containing information that could potentially be identifying have been modified without changing the sentiment. Otherwise all quotes appear with the same emphasis, spelling, grammar and use of emojis that they originally contained. No ethical approval was required for this study in accordance with King’s College London ethics for data available in the public domain.

## Results

### Introduction to dataset

Eighty-five threads matched the inclusion criteria for data analysis. Thirty-seven of these came from Mumsnet and forty-eight from Netmums. This resulted in a total of 1752 posts (range per thread: 1–206, median: 9, mean: 21). These posts were written by 956 unique usernames. Threads asking about experiences tended to contain mostly positive discussion of the topic and practical discussion about logistics, whilst threads asking for thoughts and opinions were more likely to include negative comments and arguments about the nature and ‘rationality’ of the practice. Examples of each theme brought out from the dataset can be seen in Table 1.

**Table 1:** Examples of themes

Theme	Coding frequency	Example
Positive attitudes and experiences	661	“I decided to do the placenta encapsulation as I suffered severe baby blues on the first 2... The difference seemed very noticeable, it really seemed to help me with all the the things I struggled with after the other two pregnancies“
Negative attitudes and experiences	525	“whats the views on placenta eating, be it frying it, turning it into pills or whatever, i personally dont think i could do it, the thought makes me want to heave, but each to their own. Thoughts?”
Logistical discussions	612	“Anyone tried it? If so, did your midwife/doctor help you to collect/store it? How did you prepare it for eating? Did other people e.g. your DH [dear husband] eat it too?”
Control and autonomy	98	“obviously some people have really strong views about this. .. at the end of the day,it is your choice, your placenta. .. don;t feel you shouldn;t do it because other people ridicule the idea”
Placentophagy as natural	103	“our stomachs turn quite easily in the western worldi would imagine it wouldn’t freak a lot of people out that much as a natural ritual post birth”
Placentophagy as medicine	89	“If you’re researching, go and look at actual peer reviewed studies please, not sales pitches you read online.”

### Positive attitudes and experiences

Positive comments towards placentophagy can be further divided into three broad categories; positive attitudes towards the placenta, discussion of the benefits of placentophagy, and direct positive experiences with the practice.

#### Attitudes towards the placenta

Users expressed a range of attitudes towards their placenta that informed what they planned to do with it after birth. Most commonly this was some form of awe or admiration (*N* = 73). These users described the placenta as their child’s “life support” and expressed horror at the prospect of it being treated like “medical waste” and incinerated. For instance, “my placenta kept my beautiful DD [darling daughter] alive while she was inside me, and somehow it just seemed wrong to throw into a yellow bin bag and incinerate.” Some expressed they felt conflicted in that they did not want the placenta to be disposed of but did not know what else to do with it, leading to 17 references of having a placenta in the freezer with uncertain plans for it. Users who valued the placenta as a positive part of the birth experience desired to find a way to express and honour this value. The most common suggestion given for this was to bury it (*N* = 103), which was usually perceived as something “.. .beautiful and very special to do. A treasured memory.”

#### Benefits and positives

The benefits of placentophagy appeared more frequently in discussion than any other theme (*N* = 421). The most common benefit listed was the prevention, improvement or treatment of post-natal depression (PND). This matches existing research that suggests that 73.1% of women who consume their own placenta do so to prevent PND [4]. Users expressed that they perceived placentophagy as capable of minimising other negative aspects of birth, including improving energy, improving recovery, reducing postpartum bleeding, and ensuring the ability to breastfeed. These benefits were believed to be caused by the hormones or other “nutrients” available in the placenta. It has been established that some hormones may exist in significant enough concentrations in the placenta to reach the threshold for a physiological effect *if* they could be absorbed [30]. A basic understanding of this mechanism was expressed by users, such as one who suggested birthing the placenta is “an awful lot of nutrients, iron, hormones etc. .. for the body to lose in such a relatively quick space of time and the benefits of putting that back is outstanding”.

In the discussion among users it was evident that the most common motivation for the practice was to prevent repeating a previous negative birth experience, especially in cases where the user had previously experienced PND. A typical expression was that users were willing to “try anything” to avoid it, or as one put it “Hell, if I thought cutting my foot off and eating it would help get me through PND I’d try that.” Some first-time mothers were motivated to try placentophagy due to a family history of PND or their own history of mental health problems. Previous negative experiences with being unable to breastfeed, bleeding and having low energy also motivated the practice. In total 46 users reported planning to do placentophagy with their next birth, specifically motivated by a previous negative experience. One user even explicitly said “if id had a normal birth i wdnt consider it”. This contrasts with data from the United States suggesting that placentophagy is more common in first-time mothers, although concurs that it is correlated with a personal history of anxiety or depression [4].

#### Direct experiences

Recounting a positive experience with placentophagy (*N* = 29) was much more common than reporting a negative experience (*N* = 2). Most positive experiences were contrasted to a previous birth, such as “I felt better than I had after any other birth (four).” Some went on to recommend the practice to anyone reading, whilst others were more cautious, with one user writing that they “don’t know if it had any real effect” and another concluding “May just be a coincidence but works for me!” Of the two users reporting negative experiences, one reported getting a headache alongside increased energy and lighter bleeding, describing it as “definitely effective - only not in a totally positive way in my case!” The second mentioned no benefits and complained solely about the taste after frying and eating a small amount, as “it was like eating something what ‘liver smells like.’”

### Negative attitudes and experiences

#### Attitudes towards the placenta

Eleven users expressed anger at their placenta after experiencing a difficult birth where it was retained or had to be surgically removed. After this experience they stated they hoped it had been destroyed, or that they did not want to see it again. One user had “planned to eat it” but after having their “dream home-birth ruined” by a retained placenta they let the midwife take it away – a choice they went on to regret. This example articulates how the position of placentophagy as part of the ideal birth experience changed based on the user’s attitude towards their placenta. An understanding of the placenta as a negative part of birthing appeared to lead to a desire for it to be involved in any birth plans as little as possible.

#### Risks and negative attitudes

The risks of placentophagy were mentioned four times less frequently than the benefits. Most commonly referenced was the idea that it was “unnecessary”, a scam or pseudoscientific and therefore a waste of money, rather than causing direct harm. A typical expression of this is shown by one user who wrote “Iv read nothing but good things It’s £180 if it works it’s worth the money if not then that’s a lesson learnt I suppose”. Infrequently, users expressed concerns about the sterility of the procedure and worried that the unregulated nature of encapsulation services meant that people would risk bacterial contamination for themselves or their children, especially if breastfeeding. Another concern was that if placentophagy was carried out with the desire to treat PND and it was ineffective, it could delay the individual from seeking medical treatment.

Disgust was a more common negative reaction than concern about risk. This was usually expressed by some variation of “ew”, “yuck”, or describing experiencing nausea or chills at the prospect. Some users (*N* = 16) specifically stated that it was the concept of eating something that had passed through their vagina during birth that disgusted them, usually relating to the concept of contamination by blood, bacteria or amniotic fluid. A number of users (*N* = 28) said that they believed in the benefits of placentophagy but could not bring themselves to participate due to this disgust. This was often followed by an expression of admiration or respect for women who do participate, such as “For any woman who has, you’re braver than me.”

In expressing disgust, users often related placentophagy to other products as a point of reference. This was most commonly meat (especially liver), but also other organs, faeces, urine or menstrual discharge. Additionally, comparisons were made to cannibalism, and arguments about whether placentophagy constituted cannibalism or not frequently occurred in longer threads. Users often placed the practice as adjacent to cannibalism by describing it as “virtually cannibalism”, “self-cannibalism” and “akin to cannibalism”. The comparison to other body products and cannibalism may lend itself to a discussion of where the disgust and therefore the taboo of placentophagy stems from. Field [31] suggests that systems of belief about what constitutes “clean” and “unclean” foods might be why placentophagy has not been recorded despite being nearly ubiquitous among other placental animals. She suggests that the placenta is systematically associated with birth, which is conceptualised as an “unclean” event [32]. Others have argued that the complete absence of placentophagy in the human record suggests that the taboo is more in line with behaviours that have a significant impact on an organism’s reproductive fitness [33]. This would explain why disgust occurs not just in the context of a UK parenting forum, where a cultural argument seems strongest, but throughout human history. However, the discussions captured here appear to favour an argument for taboo on the basis of cultural conventions.

Non-maternal placentophagy (where the placenta is consumed by someone other than the mother) was referenced 50 times. Users were far more negative in their responses, often directly contrasting it to maternal placentophagy. A typical response was “I can understand maybe capsules or the mother doing it but sharing it? Hell no”. This attitude lends credence to the conceptualisation of placentophagy as part of an ideal birth experience because it is *only* in relation to birth that consumption is perceived as desirable.

### Logistics and popularity of placentophagy

Logistical discussions made up a significant proportion of the discussion on threads asking for experiences or recommendations. The most commonly identified barriers to participation were the cost of encapsulation, the process of getting the placenta home from hospital, and medical contraindications to consumption. The costs discussed for paying for someone else to encapsulate the placenta ranged from £100 to £400. This led some users to ask for advice on how to do it themselves, such as this post: “.. .would be keen to hear from anyone who has done this at home - is it easy/messy/does it need a chemistry degree lol”. Users were unsure about the hospital rules about keeping their placenta, how it would be stored and whether they would be judged by staff and other patients. Users also identified several contraindications to placentophagy including blood borne viruses, staying in hospital, placental “autopsy”, calcification, and so on. Participation in forum discussion allowed users to help each other work around these barriers, and so increased the number of people for whom placentophagy was a viable option.

By far the most common method of placentophagy discussed was encapsulation (*N* = 177).^3^ The second most common was the blending of raw placenta with fruit to make a smoothie (*N* = 84). Encapsulation was viewed as more palatable and considered advantageous in that pills could be used for months after birth or saved and used during menopause. However it was also seen as costly and time consuming, usually requiring outside help. Cooking the placenta and consuming it without encapsulating it was only infrequently discussed and very rarely mentioned as a real option, more often joked about. Users expressed the belief that cooking would destroy the “nutrients” or “hormones” and therefore render the practice useless, even if it was more palatable. Users shared resources including online do-it-yourself (DIY) guides and links to encapsulation practitioners and kits. These removed some of the cost-related or knowledge-related barriers to practicing encapsulation. Resource sharing was almost always specifically in relation to encapsulation and thus encouraged the practice over other options. It is not unreasonable to assume that the increased availability of encapsulation and range of providers has led to an increase in the practice of placentophagy, given how frequent expressions of disgust were at the prospect of consuming the placenta in non-encapsulated form.

## Discussion

### What is the “ideal birth”?

This paper deals with the concept of an “ideal birth”. The ideal birth is a normative construct about what kind of birth experience women should plan for and strive to achieve. The ideal birth is part of birthing discourse characterized by two competing knowledges: the medical or technocratic view that emphasises the benefits of medicalisation and technology, and the natural or holistic view, which argues that medicalisation takes control and power away from women [34]. Pregnant people experience tensions due to the cultural pressures to conform to these competing narratives. This happens alongside pressures regarding fulfilling the social role of motherhood. These pressures feed into decisions and planning regarding parenting and pregnancy. Placentophagy is generally something decided upon before birth with the intention of improving the post-birth experience, as evidenced by the discussions captured in this paper, and is therefore understood best when examined in this context.

### Parenting forums as communities valuing experience and autonomy

Both Mumsnet and Netmums adopt a sharing and participatory ethos that is a central feature of contemporary digital communication [35, 36]. Both forums upheld atmospheres of conversation that enabled mutual support and community to be built. Topics discussed on the forums include intimate subjects such as users’ sex lives [37] and their experiences of pregnancy loss and stillbirth [38], meaning the cultural space of the forums is one that facilitates conversations that are difficult to hold elsewhere. The space is almost exclusively occupied by women (Mumsnet’s statistics report men make up 2–5% of core users [39]), and this is reinforced explicitly and implicitly by users. Brubaker and Dillaway [40] argue that parenting discussion forums are a form of “MotherSpace”, a place defined by Marotta [41] as the “discursive spaces that contemporary mothers inhabit” that “helps shape their subjectivities and their possibilities.” These forums influenced people’s decisions to participate in placentophagy by constructing it as acceptable or unacceptable behaviour.

Parenting forums also exist as a culture that prioritises the experiences of individuals over that of scientific evidence. In over half of the threads the first post explicitly asked for other users to share their experiences. It was common to acknowledge the lack of scientific evidence available in favour of placentophagy and then dismiss this as unimportant, as one user does when posting “as far as I know there is no official conclusive ‘proof’ of the benefits...not that that really matters”. Another user comments that “however there are hundreds if not 1000s of first hand experiences of women. ..” after noting a documentary found placental encapsulation was no more effective than a placebo. Previous research agrees that users on Mumsnet “constructed a collective in which personal experience was evaluated on a par with, and often favoured over, formal scientific knowledge” [29].

Users valued the individual’s right to choose to participate in placentophagy, regardless of others’ opinions. Variations of the phrase “each to their own” were common sentiments. Users would criticise other users who expressed negative sentiments without such specifications, such as “What gives anyone the right to criticise someone’s decision to keep their placenta?” In the global North mothers are performing parenthood in a sociocultural context in which there is an expectation to adhere to idealised norms [40]. In a forum populated by people who experience this burden, the commitment to not replicating these expectations allows users to share diverse experiences without fear of repercussions. This means users can discuss placentophagy (relatively) freely where they may otherwise have been, or felt, judged. Users occasionally specified that the decision was their choice because it concerned their body and their pregnancy. One wrote about their unsupportive partner “.. . it’s my body and me that is the one who gets horrific PND then I think I should get the final say in the matter..” asserting their bodily autonomy. Women can feel alienated in medical birthing settings because their knowledge of their own bodies is not considered to be authoritative [42].

### Discourse of medical and natural births

Conflicts about placentophagy were relatively uncommon and occurred mostly on threads asking for opinions on the topic. When arguments did happen, they were most commonly articulated by making references to the concepts of “natural” and “medical” births and the value of each. The distinction between the two here is that natural births are perceived to have been historically the norm and involve birthing at home or in the community with limited or no medical intervention, whilst medical births are the currently commonplace hospital-based births. Users both for and against the practice made arguments appealing to the value of both medical and natural births. The discourse produced therefore was not so much a “medical versus natural” dichotomy but rather for and against the inclusion of placentophagy in an ideal birth as articulated through medical and natural discourses. This reflects literature suggesting that very few women fully accept one of these two models and instead construct their own by negotiating ideal birth concepts with the realities of their individual experiences [43]. The following sections describe placentophagy in terms of these negotiations.

### Placentophagy as “natural”: appealing to culture, traditions and nature

Users both for and against placentophagy made appeals to culture and tradition as justification for its inclusion or exclusion from birth plans. Users for placentophagy described it as a “centuries old tradition” used in European or Chinese medicine. It should be noted that this is true, but these records relate to non-maternal placentophagy only, where someone other than the mother consumed the placenta [3]. Some users specifically identified “western” people as experiencing “peer pressure and conditioning that somehow we are above that kind of thing”, indicating their perception that placentophagy was practiced either historically or contemporaneously in non-western cultures. Users arguing against placentophagy stated in contrast that placentophagy was rare and not part of any historical or cultural tradition. Users on both sides are acknowledging that a cultural argument would be a valid argument for placentophagy, but those arguing against are denying that it fulfils the criterion.

Users from both positions also described the placenta being a convenient source of nutrition historically where other food sources were more difficult to acquire. For those against the practice, they used this position to argue that “modern” humans have access to alternative forms of nutrition in the form of food and multivitamins so there is no reason to practice placentophagy. Users arguing for the practice used it as proof that the placenta is a beneficial source of nutrients. Additionally, almost all users acknowledged that most animals consume their placentas after birth. Advocates of placentophagy argued that this meant humans should consume it as well, whilst those against the practice argued that either humans do not need the same nutritional benefit that animals gain from it or that this was done as a method of predator avoidance and therefore unnecessary. In this way users both for and against placentophagy used the concept of what is “natural” by making comparisons to history or nature and then assessed the value of “natural” births in their contemporary context.

### Medicalisation of placentophagy

Medicalisation is when a problem is defined or described in medical terms, understood through a medical framework, or “treated” with medical intervention [44]. It can be understood as a process where medical practice is used to eliminate or control experiences that are defined as deviant, in order to adhere to social norms [45]. It can be argued that placentophagy, especially placental encapsulation, has been (or perhaps always was) medicalised. It is practised to control negative birth experiences, often explicitly medical ones such as PND. It is also practised with the intention of fitting social norms such as the ability to breastfeed. Many users appealed to loosely biomedical understandings to explain the potential mechanisms for medical benefits. One user, defending concerns about bacterial contamination, wrote that they used an encapsulation provider who has “been signed off by public health England and environmental health”, ascribing the service medical legitimacy. Similarly, the Independent Placental Encapsulation Network (IPEN), often shared with other users as a resource, claims that their specialists have been approved by Brighton, Winchester, and West Hampshire Borough Councils, and that they follow a “comprehensive Food Safety Management System” [46]. An image of a pot of encapsulated placenta “pills” was posted onto one of the threads analysed. It includes “dosage” instructions and warns to “discontinue in case of infection”, mimicking medical or vitamin tablets.

Whether there is any truth to the claims made regarding benefits is uncertain but appears unlikely. Placenta is known to be a moderate source of iron but does not have any significant impact on post-partum iron status [47]. It has no effect on plasma prolactin levels or neonatal weight gain, indicating there is unlikely any improvement to breastfeeding [12]. B vitamins, beta-endorphins, prolactin, placental opioid-enhancing factor (POEF) and oestrogen have been proposed as being obtainable via placentophagy [3, 48] but a RCT found no significant differences in salivary hormone concentrations after consuming placenta compared to placebo [10] and no robust differences in postpartum mood, bonding, or fatigue [11]. It did however find a significant dose-response relationship between hormones found in the placenta and salivary hormone concentrations. An analysis of samples of placenta prepared “raw”, “steamed and dehydrated and “raw and dehydrated” found all hormones were sensitive to processing and only progesterone remained stable after dehydration, though its bioavailability is unknown [49]. Risks appear minimal; analysis of placental tissue has found trace amounts of toxic elements such arsenic, selenium and lead but these are below the toxicity thresholds for foodstuffs set by the European Union [50]. Bacterial contamination could occur, and potentially pathogenic organisms can be found in raw placental tissue, but processing appears to reduce this risk [49]. Additionally, a large medical records study including 7162 women who ate their placenta found no association with adverse neonatal outcomes, including infection [4].

The universal practice of placentophagy for perceived medical benefits distinguishes it from other practices associated with the natural birth movement (such as home births and placental burial) that are participated in from an explicit anti-medicalisation perspective [51]. Whilst Janszen concluded in [52] that placentophagy was part of the “back to nature” movement and founded on the belief that placenta consumption is common in other cultures and part of human heritage, this no longer appears to be the main motivation for the practice.

### Resisting medicalisation: placentophagy as control

It has been suggested that midwives define “natural” births differently from conventional discourse, considering a birth natural even when it involves medical interventions as long as this is something the pregnant person had been actively involved in deciding to do [53]. Brubaker and Dillaway [40] argue that this is evidence that pregnant people operationalise discourse about natural birth through notions of control and autonomy [40]. This is reflected by some users; one writes that they valued placentophagy because they “had a really traumatic, out-of-control birth with lots of intervention and this is one of the only bits I was in control of.” Another described previous medical intervention during birth in the form of a blood transfusion and writes that they “would like to try and take control this time.” There is an association made between medical intervention and lack of control, and in contrast placentophagy is repeatedly asserted as a method of control, usually when perceived as a potential solution to problems arising due a previous negative birth experience. It is known that people who become pregnant following a traumatic birth find “the emotional distress of their first experience. .. vividly reignited” [54]. To counteract this feeling some actively sought to improve their well-being through yoga, homeopathy and diets. The active attempt to improve their next birth led them to, paradoxically, accept that the outcome of it is uncertain [54]. A similar narrative is seen in the users of parenting forums who are actively trying to improve their next birth experience through placentophagy.

This is also important when we consider how parenting forums value personal choice. If medicalisation is medical authority’s usurpation of control over women’s reproduction, leading women to be required to consult medical experts for what has “traditionally and naturally” been women’s domain [40] then the return to the valuation other users’ (women’s) experiences over the medical evidence is resistance to medicalisation. Placentophagy can therefore be conceptualised as both a medicalised phenomenon and a form of resistance to medicalisation. This dual understanding exists without contradiction precisely because most pregnant people do not subscribe to a purely medical or purely natural concept of an ideal birth, instead valuing aspects of both.

### Future Research

With regards to future research indicated by this paper, it is the authors’ belief that there is not yet sufficient evidence regarding the medical risks of placentophagy to determine whether this practice should be discouraged and therefore whether there is a need to create official policy on the topic. Whilst it does not appear there is any association with adverse neonatal outcomes, this has not been studied outside of the United States. It may that be that methods and therefore frequency of contamination varies regionally. Depending on risk, it may be pertinent for hospitals to undertake a review of policy on release of placental tissue and what advice is given to pregnant people with regards to placentophagy.

### Limitations

The nature of online discussion forums means that there are inherent limitations in this research. There was no ability to determine demographic information of the users. Some of the threads contained posts that had subsequently been deleted or edited by the user or deleted by forum moderators. Users occasionally mentioned moving to direct messaging to communicate about the topic privately, and it is likely this also happened and was not mentioned. The use of parenting forums creates a self-selecting pool of participants who may have characteristics that mark them as distinct from the population of parents as whole, not least because they predominantly exclude men, so some opinions may not be heard. This study only pertains to the discussions of UK parenting forum users, and the practice in other countries may or may not be substantially different.

## Conclusions

This paper offers explanations as to the motivations for placentophagy; it is almost universally practiced for the perceived medical benefits, especially the prevention or treatment of PND. Often it is motivated by previous negative birth experiences. Potential risks were very infrequently considered, even by opponents of the practice.

There are various material reasons indicated for the increase in prevalence of placentophagy from the 1970s to present. This includes the increased resources for encapsulation, an option of consumption that reduces the seemingly-instinctual reaction of disgust to the prospect of consuming the placenta. It also includes the role of forums and other online resources in reducing logistical barriers to participating. Taking these factors into account it seems likely that placentophagy will continue to increase in popularity, especially considering the growing business for encapsulation providers.

Mumsnet and Netmums form important community spaces for mothers and pregnant women and play a role in establishing shared understandings about what is and is not acceptable birth practice. Opinions about placentophagy were vocalised through discourse on medical and natural births. Placentophagy is a practice for which there is perceived to be a large amount of anecdotal evidence but very limited scientific evidence in its favour. The experiences of other users are valued above medical authority, returning authority in birth decisions to women. At the same time placentophagy itself is medicalised, and so it is conceptualised both as a medicalised phenomenon and practiced as a form of resistance to medicalisation. This dual conceptualisation occurs because most pregnant people do not subscribe to a concept of birth that is purely medical or purely natural, and so both arguments are considered to be legitimate.

## Data Availability

As all data was obtained from publicly available archives it can be accessed by using the search terms and methods described in the methodology.

## Abbreviations

BMJ: British Medical Journal
DD: Darling/Dear Daughter
DH: Dear Husband
DIY: Do-It-Yourself
IPEN: Independent Placental Encapsulation Network
PND: Post-Natal Depression
RCT: Randomised Controlled Trial
UK: United Kingdom
US: United States

## Footnotes

1 Found at: https://www.mumsnet.com/Talk and https://www.netmums.com/coffeehouse/ respectively [accessed 30/05/2018].

2 Netmums siteowner Sally Russell reported in 2006 that 40% of users were from low income families [25]. She further reported in 2012 that 19% of users had household incomes of less than £15,000 and 10% between £15,000 and £20,000 [26], which are considerably lower than the UK average household income of £28,400 [27].

3 There are two main methods of encapsulation, the difference between them being whether the placenta is steamed or not before being dehydrated, pulverized and encapsulated. Steamed encapsulation is also known as the “Traditional Chinese Method” (TCM), whilst non-steamed encapsulation is often referred to as “raw encapsulation”. Users of these forums largely did not specify which kind of encapsulation they were referring to.
